# Successful sequential tyrosine kinase inhibitors to overcome a rare compound of EGFR exon 18–18 and EGFR amplification: A case report

**DOI:** 10.3389/fonc.2022.918855

**Published:** 2022-07-25

**Authors:** Pascal Wang, Emmanuelle Fabre, Antoine Martin, Kader Chouahnia, Ambre Benabadji, Lise Matton, Boris Duchemann

**Affiliations:** ^1^ Thoracic and Medical Oncology Department, Avicenne Hospital, Assistance Publique-Hôpitaux de Paris (AP-HP), Bobigny, France; ^2^ Biochemistry Department, Avicenne Hospital, Assistance Publique-Hôpitaux de Paris (AP-HP), Bobigny, France; ^3^ INSERM UMR 978, Sorbonne Paris Nord University, Bobigny, France; ^4^ Department of Pathology, Avicenne Hospital, Assistance Publique-Hôpitaux de Paris (AP-HP), Bobigny, France; ^5^ Laboratoire d’immunomonitoring en Oncologie, CNRS-UMS 3655 and INSERM-US23, Villejuif, F-94805, Gustave Roussy Cancer Campus, Villejuif, France

**Keywords:** NSCLC, uncommon mutation, exon 18, tyrosine kinase inhibitor (TKI), compound mutations, EGFR

## Abstract

**Background:**

New mutational detection techniques like next-generation sequencing have resulted in an increased number of cases with uncommon mutation and compound mutations [3%–14% of all epidermal growth factor receptor (EGFR) mutations]. In rare exon 18 mutations (3%–6%), G719X and E709X represent the majority, but CMut associating these exon 18 points mutations are even rarer, making the understanding of the impact of epidermal growth factor receptor tyrosine kinase inhibitors still limited. Three generations of EGFR tyrosine kinase inhibitors (TKIs) are available to target EGFR mutations, but according to the types of mutations, the sensitivity to TKI is different. Afatinib, osimertinib, and neratinib have showed some effectiveness in single exon 18, but no report has precisely described their efficiency and acquired mechanism of resistance in a CMut of exon 18–18 (G719A and E709A).

**Case presentation:**

We report a case of a 26-year-old woman with bilateral advanced adenocarcinoma of the lung harboring a compound mutation associating G719A and E709A in exon 18, who developed an EGFR amplification as resistance mechanism to osimertinib. She presented a significant clinical and morphological response under sequential TKIs treatment (afatinib, osimertinib, and then neratinib).

**Conclusion:**

A non-small cell lung cancer (NSCLC) with rare compound mutation exon 18–exon 18 (G719A and E709A) and EGFR amplification can be overcome with adapted sequential second- and third-generation TKIs. This report has potential implications in guiding decisions for the treatment of these rare EGFR mutations.

## Introduction

Activating epidermal growth factor receptor (EGFR) driver mutations can occur in exons 18–21, and thanks to the development of new techniques of massive sequencing [like next-generation sequencing (NGS)], more than 600 variants of EGFR mutations have been discovered so far. These mutations are classified according to their epidemiological frequencies. On the one side are “common” mutations representing approximately 80% of cases that are constituted by deletions in exon 19 (del 19) and the point mutation L858R in exon 21. On the other side are “rare” or “uncommon” mutations (Umut) with points mutations G719X of exon 18, S768I of exon 20, L861X of exon 21, and insertions/deletions/duplications in exon 20, which represent <20% of the cases. Concerning response to treatment, “common” mutations respond slightly better to EGFR tyrosine kinase inhibitors (TKIs) (1, 2). Exon 18 mutations (detected in 3.6% of all) are mainly represented by the G719X and E709X mutations (3). However, Umut and rare mutations are not fully characterized in their clinical significance and implications, even more when they are associated with each other to form compound mutations (Cmut) (4). Cmut are defined as mutations combining two or more EGFR mutations; they often consist (but not always) of a “common” mutation associated with a “rare” or a “very rare” mutation (2).

To our knowledge, we present here the first case of non-small cell lung cancer (NSCLC) with a rare compound mutation of exon 18–18 (G719A and E709A) treated by a successful sequential use of TKIs (afatinib, osimertinib, and neratinib) and developed an EGFR amplification as mechanism of resistance under osimertinib.

## Case report

In January 2016, a 26-year-old Caucasian woman was admitted to the Medical Oncology Department at Avicenne Hospital due to the discovery of a suspicious lung mass in the left lower lobe with bilateral lung lesions on a contrast-enhanced computed tomography (CT) scan performed in the context of low abundance hemoptysis and cervical lymphadenopathy. The patient was healthy in the past, a former light smoker (11 pack-years), and had no disease history reported. Because she had no case of family cancer, germline mutation was not sought. A cervical lymph node biopsy was performed by ultrasound-guided biopsy. The cell morphology was consistent with lung adenocarcinoma, and the tumor cells were positive for thyroid transcription factor-1 (TTF-1). After an extension assessment, the patient was diagnosed with a metastatic lung adenocarcinoma (cT3N3M1a, stage IV A). To clarify the genetic alteration of the tumor, molecular analysis by next-generation sequencing (NGS) of DNA (Tumor Hot Spot MASTR Plus, Multiplicom) was performed on the cervical lymph node biopsy and showed 2 points mutations in EGFR exon 18, namely, p. G719A (C.2156G>T) and p. E709A (C 2126A>C). Immunohistochemistry highlighted a MET hyperexpression (3+) but without MET amplification [fluorescence *in situ* hybridization (FISH), ratio c-MET/chromosome 7 = 2.62]. No other molecular alterations were found.

**Figure 1:**
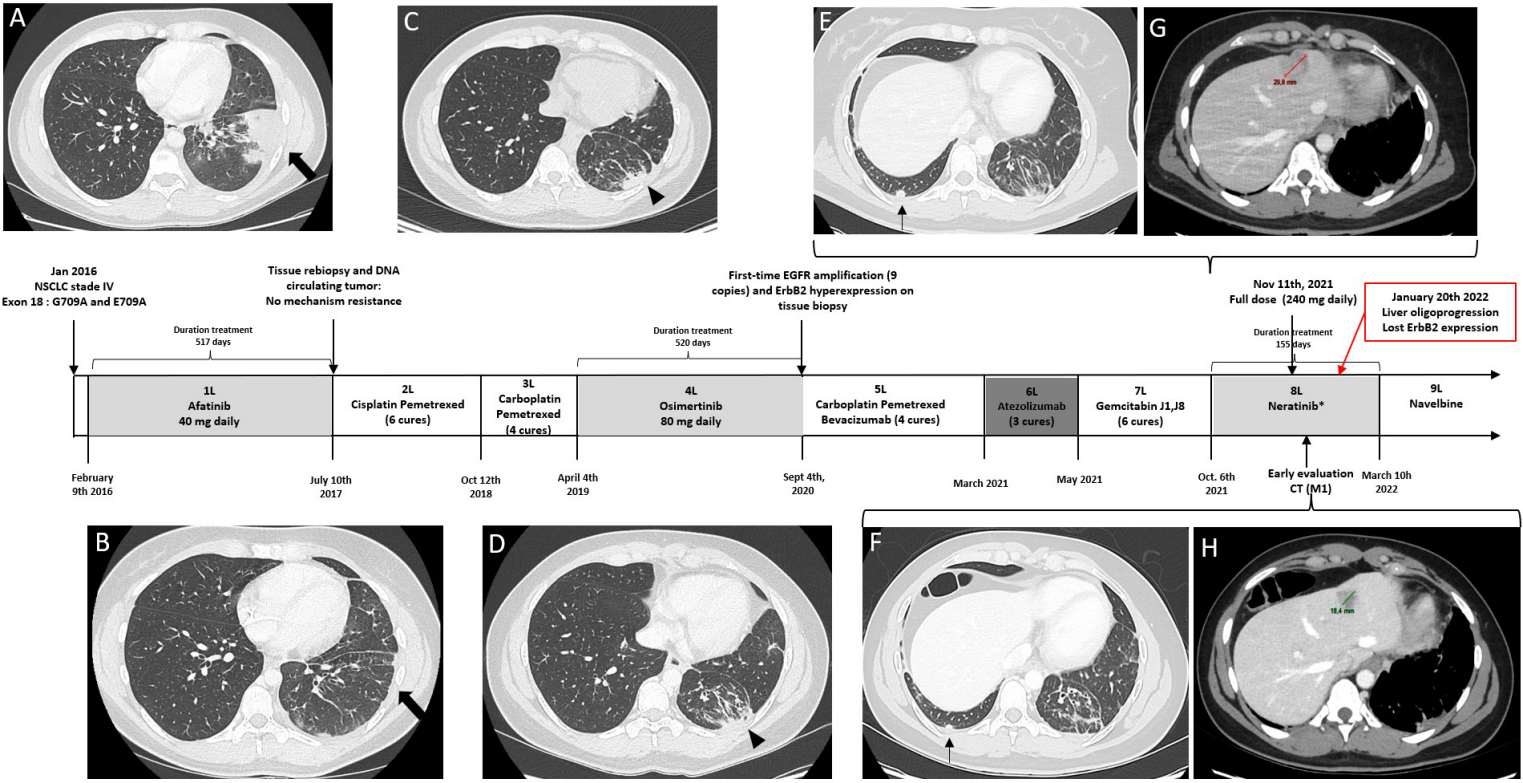
Timeline summary with corresponding CT of the different therapeutic lines between February 2016 and November 2021. Baseline chest CT showing **(A)** a lung mass in the left lower lobe. Best response under afatinib with a partial response (**B**, thick arrow). Chest CT before osimertinib’s beginning **(C)** and follow-up CT chest with a stable disease as best response (**D**, arrowhead). Baseline chest CT **(E)** and contrast-enhanced liver **(G)** CT before neratinib. At 1 month, early assessment showed a partial response of all lung lesions (**F**, thin arrow) and liver lesions (**H**, a target lesion, 29.8 vs. 18.4 mm, in the longest axis). Red arrow and case: the liver’s oligoprogression at second CT evaluation under neratinib and a new liver biopsy was done. Immunohistochemistry showed the loss of ErbB2 hyperexpression. CT, computed tomography.

Afatinib (40 mg daily) was started as first-line treatment based on the UMut. After 2 months, CT showed a partial response (−75%), and she presented a clinical improvement but suffered from a grade 2 (CTCAE v5.0) skin rash, which was relieved by doxycycline. The clinical and morphological response was confirmed for 13 months (**Figures 1A, B**). In June 2017, faced with an asymptomatic progression on lung lesions and mediastinal lymph node, NGS of circulating DNA (Kit Oncomine Focus Assay) and targeted rebiopsy were done but found no resistance mutations, especially T790M. Several combinations of chemotherapy were prescribed: cisplatin-pemetrexed [progression-free survival (PFS), 15 months] then carboplatin-pemetrexed (PFS, 6 months).

In April 2019, a relapse of thoracic and spleen lesions occurred. Hence, after a molecular board discussion, she received osimertinib (80 mg daily), which allowed an improvement for 16 months until reassessment CT revealed a pulmonary progression. The best morphological response was a stable disease (**Figures 1C, D**). Osimertinib’s plasma concentration monitoring was within standards (231 ng/ml). No mechanism of resistance (T790M and C797S) was found on liquid biopsy, but NGS (Panel Oncomine Comprehensive Assay v3) of new biopsy on lung lesions revealed the same CMut (p.G719A and p.E709A) and a new EGFR amplification (nine copies). Immunohistochemistry highlighted an intermediate ErbB2 hyperexpression (2+) without MET hyperexpression.

From September 2020 to July 2021, she received carboplatin–pemetrexed–bevacizumab (PFS, 5 months), atezolizumab (PFS, 2 months), and gemcitabine (PFS, 3 months).

On 6 October 2021, the patient received neratinib on a compassionate-based access. The TKI was started at 40 mg daily, with a gradual increase of 40 mg/week for a final dose of 240 mg daily; full dose was obtained on 11 November. Early assessment by CT at 4 weeks showed a partial response (−38%) with a decrease in all pulmonary (**Figures 1E, F**) and liver lesions (**Figures 1G, H**). She experienced a clinical improvement but suffered from a grade 1 diarrhea. The response was maintained until 20 January 2022, when a dissociated hepatic progression on pre-existing lesions is objectified on the second CT evaluation. At this time, a liver biopsy was performed, followed by a radiofrequency ablation of these lesions while continuing neratinib. On 10 March, a new CT reassessment shows new hepatic and thoracic lesions, corresponding to a clear progression. Molecular analysis on the liver biopsy by NGS (Kit Oncomine Focus Assay) of DNA and RNA found the same CMut of exon 18 and an EGFR amplification without other targetable molecular alteration. Immunohistochemistry found a PD-L1 rate of 50% but no longer found HER2 overexpression. Considering this information, a switch to weekly intravenous navelbine was started.

## Discussion

The literature showed that the response to TKIs depends on mutations that form the compound. For instance, in tumors with a CMut including exon 19 deletion or L858R point mutation, Hata et al. showed that they had the same response rate to EGFR-TKI as those with the same mutation alone (5). Therefore, in CMut with common mutations, it is recommended to start osimertinib as a first-line treatment.

For tumors with uncommon CMut, considering the composition of CMut seems also effective. Indeed, for first-generation EGFR-TKI, Chiu et al. demonstrated that patients harboring uncommon CMut (without classical mutations and at least one exon 18 mutation) had a longer PFS than patients with a single exon 18 mutation G719X (11.9 months vs. 6.5 months) (6). This trend is confirmed by Passaro et al. who showed that patients under first- or second-generation TKI with CMut (with an exon 18 mutation) had superior efficacy than patients with a single exon 18 in regard to median overall survival (OS) [hazard ratio (HR), 0.62; 95% CI (0.39–1.00)] (7). Therefore, the question is to know which EGFR-TKI is the most efficient in a CMut exon 18–18.

A *post-hoc* analysis of prospectively collected data from LUX trials showed clinical activity of afatinib in advanced NSCLC harboring UMut (G719X, L861Q, and S768I) (8). Indeed, Yang et al. reported a 71% overall response rate (ORR) and 11 months PFS in this UM. Even better, they found an increase in ORR of 77.8% and PFS of 13.8 months in the G719X subgroup. Hence, afatinib obtained approval by the US Food and Drug Administration (US-FDA) for patients with metastatic NSCLC harboring UMut and by extension, CMut with UMut.

Recently, a multicenter phase II trial (KCSG-LU15-09) demonstrated that osimertinib also has an activity in patients harboring UMut. In this study, osimertinib was given as a front line in 22 cases (61%), and mutations were predominantly G719X (n=19) and L861Q (n=9). Within G719X mutations, four were CMut (two G719 X + L861Q and two G719X + S768I), but none has a CMut exon 18–18, ORR was 53% [95% CI (28%–77%)], and mPFS was 8.2 months [95% CI (6.2–10.2)] (9).

Based on these results, we can say that tumors with a CMut have a preserved sensitivity to afatinib (but lower than common mutation) and that osimertinib is also a good choice in the context of a CMut, even without T790M. Although cross trial comparisons should be performed with caution, it seems more prudent to start with afatinib than osimertinib in view of better results in LUX trials than in KCSG-LU15-09. However, it is important to note that in the *post-hoc* analysis from LUX trials, no information was given concerning the patient’s brain status, whereas KCSG-LU15-09 reported 25% patients with brain metastases who could potentially explain this difference. In our case, the patient was free from any cerebral lesions since the beginning, which supports the choice of afatinib before osimertinib.

Moreover, Kobayashi et al. have investigated *in vitro* the sensitivities to three generations of EGFR-TKIs in retrovirally transfected cells that harbor exon 18 (including G719X and E709X) and del 19 mutations. They found that IC_90_ of first- and third-generation TKIs in exon 18 mutations were much higher than those in del 19 (by >11–50-fold), whereas IC_90_ of afatinib were only three- to seven-fold greater than del 19 (3). Therefore, *in vitro* and *in vivo*, second-generation TKI seems more efficient than first or third generations in exon 18 mutation and, by extension, in CMut with exon 18 mutation.

Neratinib is an irreversible pan ErbB (EGFR/HER2/HER4) oral inhibitor, first studied by Sequiest et al. in 2010, who have already demonstrated neratinib efficiency in some patients harboring G719X mutation (10). At this time, it had been more studied for its properties to overcome T790M mechanism resistance than for its high sensitivity to exon 18 mutation. In 2021, the preliminary result of SUMMIT (n=11) confirmed the neratinib efficiency in exon 18 and demonstrated an ORR of 36% [95% CI (11–69)] and a PFS of 6.9 months. Moreover, among them, one patient had the same CMut exon 18–18 as in our case report (G719A and E709A) and presented a partial response lasting for 16 weeks (11). In view of these encouraging results from the SUMMIT trial and the presence of ErbB2 overexpression under osimertinib, neratinib seemed to be the best therapeutic alternative to target at the same time the CMut exon 18–18 and the ErbB2 hyperexpression.

There are limitations to data about sequential treatments for uncommon exon 18 mutation. Indeed, in the KCSG-LU 15-09 trial (9), one of the exclusion criteria was previous treatment with EGFR-TKI, and in the SUMMIT trial (11), 91% (10/11) patients have prior EGFR-TKI, but they have no clue regarding these therapeutic sequence and resistance mechanism prior to EGFR-TKI.

The strength of our case is the precise description of a workable therapeutic sequence of TKIs that allowed prolonged response, causing a dramatically improved OS of an exon 18 mutation and EGFR amplification. Indeed, EGFR amplification is a usual acquired EGFR-dependent mechanism after osimertinib in first (~10%) or second (6–10%) line for patients with common mutations but not yet described in the setting for CMut exon 18–18 (12, 13).

Even more, this case is interesting because she also had a moderate ErbB2 hyperexpression found at progression on osimertinib, which is known to potentially lead to a decreased sensitivity to osimertinib (14). Therefore, the osimertinib resistance mechanism is potentially heterogeneous with association of several resistance mechanisms usually found in common mutation. This hypothesis is reinforced by the fact that the liver’s oligoprogression under neratinib was accompanied by the disappearance of ErbB2 hyperexpression, which had probably contributed to an initial and brief sensitivity to the pan ErbB, neratinib.

Concerning alternatives after navelbine, antibody–drug conjugate (patritumab–deruxtecan or HER3-DXd) and bi-specific antibodies (amivantamab) seem to be particularly interesting, especially since these molecules are currently tested in situations of osimertinib failure (15, 16). These antibody drugs are even more attractive, as they have been tested in situations where the resistance mechanisms were not clearly defined. Indeed, Jänne et al. showed recently in a phase II trial that patritumab–deruxtecan was efficient in EGFR-mutated NSCLC heavily pretreated, regardless of the brain status, the resistance mechanism identified, and the level of HER3 expression. They had received, in median, four lines [1–9] of treatment before, including osimertinib (89%), a platinum doublet (80%), and immunotherapy (35%). The objective response rate was 39% [95% CI (26.0–52.4)], the control rate was 72% [95% CI (58.5–83)], and the median progression-free survival was 8.2 months [95% CI (4.4–8.3)] (15). In this case and given the limited state of knowledge on the mechanism of resistance and the sensitivity of TKIs in rare CMut, theses antibody therapies have a place of choice.

Furthermore, it should not be forgotten that a retreatment effect linked to prolonged periods of “TKI holidays” is also possible in our clinical case. Indeed, the effect of retreatment is well described in common mutations (17). It is based on the fact that after one or more lines of TKI, the resistance mechanisms are often heterogeneous and multiple in the same patient (20%–50% of patients according to studies that analyzed resistance mechanisms by circulating tumor DNA after a third-generation TKI) (18, 19). Indeed, these patients will present different subpopulations of cancer cells with heterogeneous mutations, which can be potentially insensitive to EGFR-TKI. The prescription of chemotherapy following EGFR-TKI would make it possible to target all subpopulations and thus reduce the proportion of resistant clones and then to restore sensitivity to TKI by promoting the re-emergence of clones with a targetable mutation (20). This principle was well described in the study by Ichihara et al. who retrospectively studied the rechallenge of osimertinib after chemotherapy treatment in 15 patients who relapsed after osimertinib. During osimertinib rechallenge, the authors found an ORR of 33%, a disease control rate (DCR) of 73%, and a PFS with 4.1 months, which showed a resensitization to osimertinib. Thus, one of a possible confounding factor in our case is the prescription of chemotherapy between each line of TKI, whose periods could go up to 9 months. Nevertheless, the effect of retreatment is not yet known in UMut and particularly in rare CMut.

As a conclusion, this case emphasizes the potential benefit of sequential TKI therapy of second followed by third generation despite the absence of T790M mutation in patients with UMut. Resistance to third-generation EGFR-TKIs may involve EGFR amplification and probably ErbB2 hyperexpression in CMut exon 18–18 and can be treated by a pan ErbB inhibitor. Reports of these rare compound mutations, mechanisms resistances, and their response to TKIs are necessary to improve knowledge of this UMut. However, more research is needed before neratinib can be recommended as a new standard in CMut exon 18–18.

## Data availability statement

The raw data supporting the conclusions of this article will be made available by the authors, without undue reservation.

## Author contributions

Conceptualization: PW and BD. Methodology: BD. Investigation: PW, BD, EF, and AM. Ressources: PW. Supervision: BD. Validation: PW, EF, AM, KC, AB, LM, and BD. Visualization: PW and BD. Writing – original draft: PW and BD. Writing – review & editing: PW, EF, KC, LM, and BD. All authors contributed to the article and approved the submitted version.

## Conflict of interest

BD: Payment or honoraria for lectures, presentations, speakers bureaus, manuscript writing or educational events: Pfizer, Roche, Chiesi. Payment for expert testimony: AstraZeneca, BMS.

The remaining authors declare that the research was conducted in the absence of any commercial or financial relationships that could be construed as a potential conflict of interest.

## Publisher’s note

All claims expressed in this article are solely those of the authors and do not necessarily represent those of their affiliated organizations, or those of the publisher, the editors and the reviewers. Any product that may be evaluated in this article, or claim that may be made by its manufacturer, is not guaranteed or endorsed by the publisher.
